# Short-term temperature fluctuations show minimal effects on worker gut microbiota in *Bombus terrestris* under controlled conditions

**DOI:** 10.3389/finsc.2026.1867923

**Published:** 2026-08-28

**Authors:** Nuria Blasco-Lavilla, Andrés García-Reina, Alejandro López-López, Pilar De la Rúa

**Affiliations:** 1 Department of Zoology and Physical Anthropology, Faculty of Veterinary, University of Murcia, Murcia, Spain; 2 Department of Invertebrate Evolution, Institute of Zoology and Biomedical Research, Faculty of Biology, Jagiellonian University, Kraków, Poland

**Keywords:** bumblebee, climate change, colony thermoregulation, microbiota, temperature, thermal stress

## Abstract

Insect symbionts are key contributors to many physiological traits of their hosts. The bumblebee microbiota is mainly composed of a few taxa that, although not necessary for their survival, present a long history of coevolution with their hosts, and contribute to their nutrition and defense against pathogens. Mutualistic interactions with endosymbionts can be affected by environmental factors, such as temperature. Bumblebees have the particularity of thermoregulating their nests, which might help the maintenance of these mutualisms. The aim of this study was to analyze how environmental temperature stress affects bumblebee’s microbiota composition both within the nest environment and without the effect of colony thermoregulation. We exposed *Bombus terrestris* workers to environmental temperatures of 9 or 38 °C (within the range of temperatures of the species habitat) for five days, both within their original colonies and in small microcolonies. Then, we returned all workers to 28°C (control temperature) for five additional days to study if microbiota composition could be restored after thermal stress in case of changes. Analyses of microbiota composition indicated that temperature treatment had a significant effect on the workers, whereas the colony environment had a significant effect on the workers after the recovery period. *Post-hoc* analyses indicated that the heat treatment had a greater effect on microbiota composition than the cold treatment, but none of them were significant. Our results point to a robustness of bumblebee microbiota to temperature changes. Bumblebee symbionts could have adapted to temperature changes as a result of their exposure to very variable temperatures during queen hibernation, foraging, brood incubation and *ex vivo* phases outside nests.

## 1 Introduction

Insect guts harbor microorganisms that are often beneficial to the host. In recent years, insect gut microbiota has been revealed to play an important role in many aspects of their physiology such as nutrition, defense against pathogens, removal of toxins (1), development, reproduction (2), and thermal tolerance (3). On the other hand, environmental factors that affect microbial activity such as diet, pH or temperature can indirectly impact host health via perturbation to the microbiome.

As a consequence of climate change, both mean global temperature and the incidence of transient extreme temperatures are expected to increase (4). Insects are ectothermic animals whose body temperature and physiology depend on environmental fluctuations. Therefore, mutualistic associations with microorganisms with narrow thermal tolerance can limit host adaptation to environmental change (3, 5). This is the case of obligate symbionts, where long coevolution has resulted in bacteria being required for normal function of host insects and the degenerative genome evolution of the symbiont to preserve only essential genes, relying on the host for survival. Consequently, obligate symbionts lose evolutionary potential and accumulate deleterious mutations that translate to proteins with less thermal tolerance (5, 6). Both factors result in obligate mutualisms being especially vulnerable to environmental thermal changes.

The thermal sensitivity of insect mutualistic interactions has been already shown in several insect orders. Maintenance of stinkbugs at 30°C caused the reduction and even loss in the second generation of gut symbionts that were required for host survival at 23°C (7, 8); heat treatment for four weeks depleted an obligate symbiont by 99% in two ant species (9) and continuous exposure to 35°C almost completely depleted the symbionts residing within bacteriocytes in the whitefly *Bemisia tabaci* (10). Moreover, bacteria from the lineages *Firmicutes* and *Proteobacteria* have a tendency to decrease and increase their relative abundance respectively with higher temperatures and across different host species (11).

To date, there is limited information on how temperature can affect bee microbiota despite the large evidence of decline in several bee species (12). Social bees have a highly host-specialized gut microbiota composed of five core clades: *Gilliamella, Snodgrassella, Lactobacillus, Bombilactobacillus* and *Bifidobacterium.* They support their host nutrition (13–15) and defense against pathogens (14, 16) although they are not necessary for their host’s survival (17). These bee symbionts are transmitted via fecal-oral route mainly between nest-mates (18, 19) and some of them are restricted to bee guts (20).

Social bees can regulate their body and nest temperature (21) providing a stable environment for the resident bacteria. In addition to the insulation that the nest provides, social bees have different behaviors to actively maintain nest temperature such as clustering around the brood and generating metabolic heat, fanning warm air away from the nest, and spreading water for evaporative cooling (22). However, despite the relatively stable temperature of nests, symbionts still experience periods of fluctuating temperatures in the gut of foraging bees and hibernating bumblebee queens, or between periods of horizontal transmission when they are found *ex vivo* (23).

Several field studies have explored how the honey bee microbiota varies seasonally with different results (24–32). However, variables like individuals’ age, diet changes, possible infection by pathogens and behavioral changes make it especially difficult to evaluate the effect of temperature (28). Despite this, Ludvigsen et al. (29) found that the midgut/pyloric microbiota remained stable from November to February with marked environmental temperature changes possibly due to the stability of the colony environment. In bumblebees, Bosmans et al. (33) found that the midgut and ileum of *Bombus terrestris* queens exhibited decreased levels of some core bacteria and increased relative abundances of non-core bacteria after hibernation. Similarly, Hotchkiss et al. (54) observed a decrease in total microbial abundance and were unable to detect *Schmidhempelia* and *Snodgrassella* phylotypes in the guts of *Bombus impatiens* queens during late diapause. A study on parasitic infection and gut microbiota dependence on temperature in *Bombus impatiens* reported that Orbaceae and Neisseriaceae members moderately declined with temperature and Lactobacillaceae members increased twofold along the range from 21°C to 37°C (34). Finally, previous studies on thermal tolerance of bee symbionts have observed that *Gilliamella*, *Snodgrassella* and *Lactobacillus* strains can still grow at 40°C and may have adapted to the thermoregulated bodies of the bees (23, 35, 36).

The aim of this study was to test the effect of temperature on bumblebee gut microbiota and how colony thermoregulation can buffer the potential impact of temperature on microbiota composition. To do so, we exposed workers from the species *Bombus terrestris* at different temperatures: cold (9°C), heat (38°C) and control (28°C) within their original colonies and in small microcolonies. These temperatures are well within the range of temperatures of the species’ habitat (37). Including control colonies and microcolonies helps to distinguish the effect of thermoregulation from other colony-related effects, such as microbial transmission among nestmates. Additionally, stressed workers were returned to control temperature (28°C) to assess if the microbiota can be reestablished after the potential impact of mild temperature stress. We worked with the following hypotheses:

1. Hot and cold temperatures alter worker gut microbiota. Additionally, taking into account the heat tolerance of some symbionts, cold temperatures are expected to have a higher impact.
2. The impact of hot and cold temperatures on gut microbiota composition is lower in workers within colonies than in workers within microcolonies, due to the colony thermoregulation effect.
3. The gut microbiota recovers its original composition when workers within colonies are returned to 28°C. However, workers in microcolonies might permanently lose some bacterial operational taxonomic units (OTUs) as a consequence of temperature stress and limited contact with colony members and contaminated colony surfaces.

## 2 Materials and methods

### 2.1 Bumblebee treatments

Six young colonies of *B. terrestris* were obtained from Agrobio (Almería, Spain) and maintained at 28°C and 60% humidity for 10 days. Two groups of four random workers per colony were collected into microcolonies to test a possible buffering effect of colony thermoregulation on the microbial communities of workers exposed to temperature stress conditions. Colonies 1 and 2 and their respective microcolonies, were exposed to heat (38°C), colonies 3 and 4 and their microcolonies were kept as controls (28°C), and colonies 5 and 6 and their respective microcolonies, were exposed to cold (9°C). After five days all the colonies and microcolonies were returned to 28°C to check for microbiota reestablishment. Six individuals per colony and condition were sacrificed before starting the different temperature treatments (T0), after five days of temperature stress treatments (T1) and after five days of recovery at 28°C (T2). Bees were preserved in ethanol at -80°C until they were analyzed.

### 2.2 DNA extraction and amplification

Worker guts were extracted aseptically for DNA extraction. DNA was extracted using the Dneasy Blood and Tissue Kit (Qiagen), following manufacturer’s instructions. The region V3-V4 of the 16S rRNA gene was amplified from DNA samples using broad-coverage primers (38). To increase amplification efficiency, a first PCR was performed using these primers with individual samples. PCR amplicons from worker guts belonging to the same colony and condition were pooled before performing the second PCR including Nextera Transposase adapter sequences and a final PCR using Nextera XT Index Kit v2 (Illumina) primer set. The program for the first and the second PCR consisted in an initial denaturation at 94°C for 3 min, cycles of denaturation at 94°C for 30 s, annealing at 53°C for 40 s and elongation at 72°C for 1 min, and a final elongation at 72°C for 5 min. 28 and 15 cycles of amplification were used for the first and the second PCR, respectively. The program for the third PCR consisted in an initial denaturation at 94°C for 3 min, 8 cycles of denaturation at 95°C for 30 s, annealing at 55°C for 30 s and elongation at 72°C for 30 s, and a final elongation at 72°C for 5 min.

### 2.3 Bioinformatic analysis

Amplicons were generated in a MiSeq Illumina platform (2 x 350 bp). Raw sequences were processed according to the instructions available at Creedy et al. (39): demultiplexing and primer removal were performed using the software cutadapt (40), pair merging with PEAR (41), and quality filtering, denoising and chimera removal with VSEARCH (42). Sequences were clustered into OTUs with 99% similarity. Only OTUs represented by more than 0.5% of the reads within each sample were retained. Taxonomic identifications were assigned to OTUs using the BEExact database (43) with the IDTAXA algorithm (44) and a confidence threshold of 50, using the *DECIPHER* R package (45).

OTU abundance and taxonomy tables were analyzed with R 4.1.1 (62) using the packages *ggplot2* (46), *MASS* (47), *microbiome* (48, *phyloseq* (49) and *vegan* (50). Rarefaction curves were constructed to verify that the sequencing was deep enough to capture strain diversity. Alpha-diversity was estimated within each group using the Shannon index. For beta-diversity analyses, read counts were centered log-ratio (CLR) transformed using the ‘transform’ function of the *microbiome* package (48). Zero handling was performed automatically by the *microbiome* package, which applies to a pseudo count equal to half of the minimum observed relative abundance value to exact-zero entries in the OTU table prior to log transformation. (Lahti & Shetty, 2018). A two-way permutational analysis of variance (PERMANOVA) was performed using the ‘adonis’ function of the *vegan* package on the control group samples, with colony environment and time of collection as factors, to discard a possible effect of these variables on gut microbiota composition. In order to test the effect of temperature, recovery and the colony thermoregulation effect on gut microbial communities, a PERMANOVA was performed using the samples collected at the second and third time points, with temperature treatment and the colony environment as factors. We tested the assumption of homogeneity of multivariate dispersions before conducting the analyses. If a significant effect of temperature was found, a *post-hoc* analysis was carried out using the package *pairwiseAdonis* (51) to find whether the cold or the heat treatment had a significant effect on gut microbiota composition. Principal component analyses (PCA) were performed on CLR-transformed data using the ‘ordinate’ function from the *phyloseq* package (method = ‘RDA’, distance method = ‘euclidean’) to visualize variation on microbiota composition between different temperature and colony settings. The Analyses of Compositions of Microbiomes with Bias Correction (*ANCOMBC* R package, 52) was used to find differentially abundant OTUs among treatments.

## 3 Results

### 3.1 Bacterial sequences and classification

**Figure 1:**
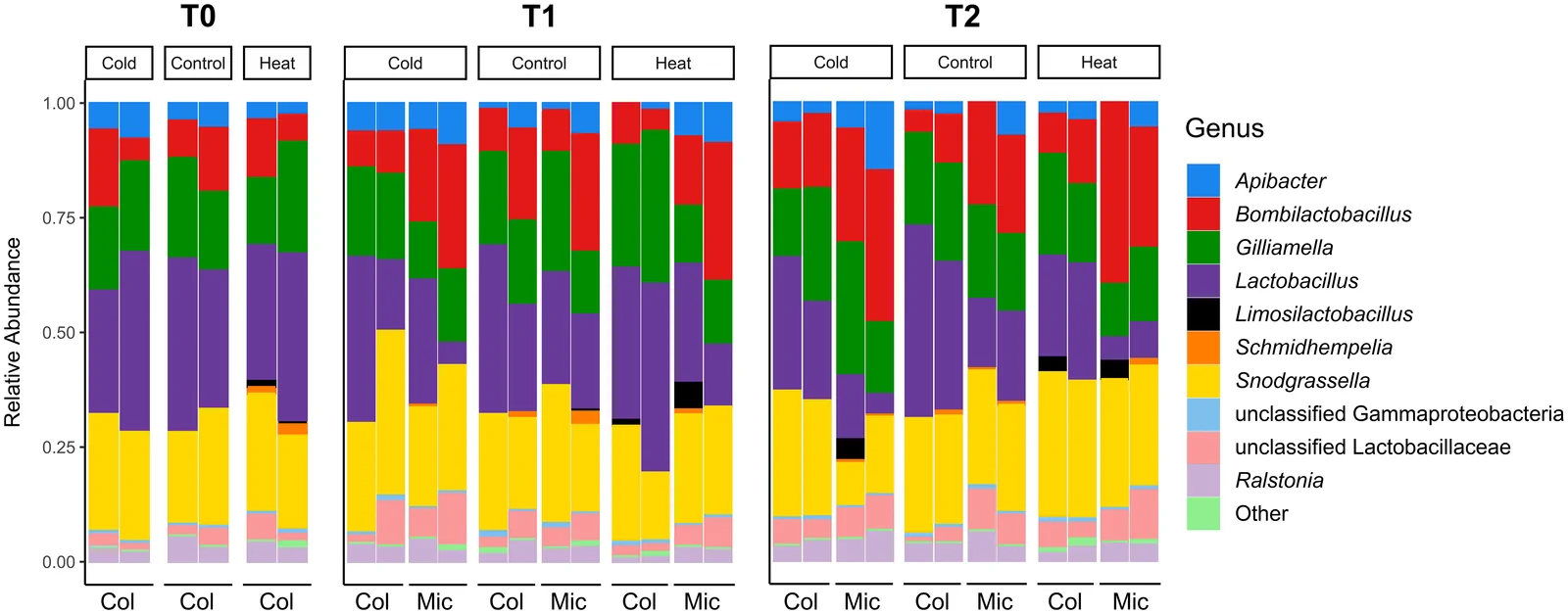
Barplots with compositional data of the gut microbial communities of workers in different temperature and colony settings. Gut communities were dominated by the families Lactobacillaceae (genera *Bombilactobacillus, Lactobacillus* and *Limosilactobacillus*), Neisseriaceae (genus *Snodgrassella*), Orbaceae (genus *Gilliamella*), Weeksellaceae (genus *Apibacter*) and Burkholderiaceae (genus *Ralstonia*). “Other” refers to genera present in less than 0.5% of reads in the whole dataset. T0: time of collection before temperature stress treatments, T1: time of collection after five days of temperature stress treatments (9°C, 28°C and 38°C), T2: time of collection after five days of recovery at 28°C, Col: colony environment; Mic: microcolony environment.

Illumina reads are deposited in the NCBI Sequence Read Archive (SRA), under the BioProject ID PRJNA1095982. The final dataset from 16S rRNA gene sequencing contained 558,906 reads mapped to 60 OTUs (**Supplementary Files**). Rarefaction confirmed that sequencing of pooled samples was deep enough to reveal the community diversity (**Supplementary Figure S1**). The gut bacterial communities were mainly composed by the families Lactobacillaceae (46.7%), Neisseriaceae (23.9%), Orbaceae (19.9%), Weeksellaceae (4.7%) and Burkholderiaceae (4%) (**Figure 1**). Reads from the Burkholderiaceae family corresponded to a strain of *Ralstonia pickettii*. This species is common as a laboratory contaminant (53) and comprised more than 99% of the reads in negative DNA controls. Notably, OTUs classified as Bifidobacteriaceae, including two classified at the genus level as *Bifidobacterium*, a recognized core member of the bee gut microbiota (20), were present in the raw dataset but fell below the 0.5% relative abundance threshold and were therefore excluded from downstream analyses.

### 3.2 Effect of temperature stress and the colony environment on gut microbiota composition

**Figure 2:**
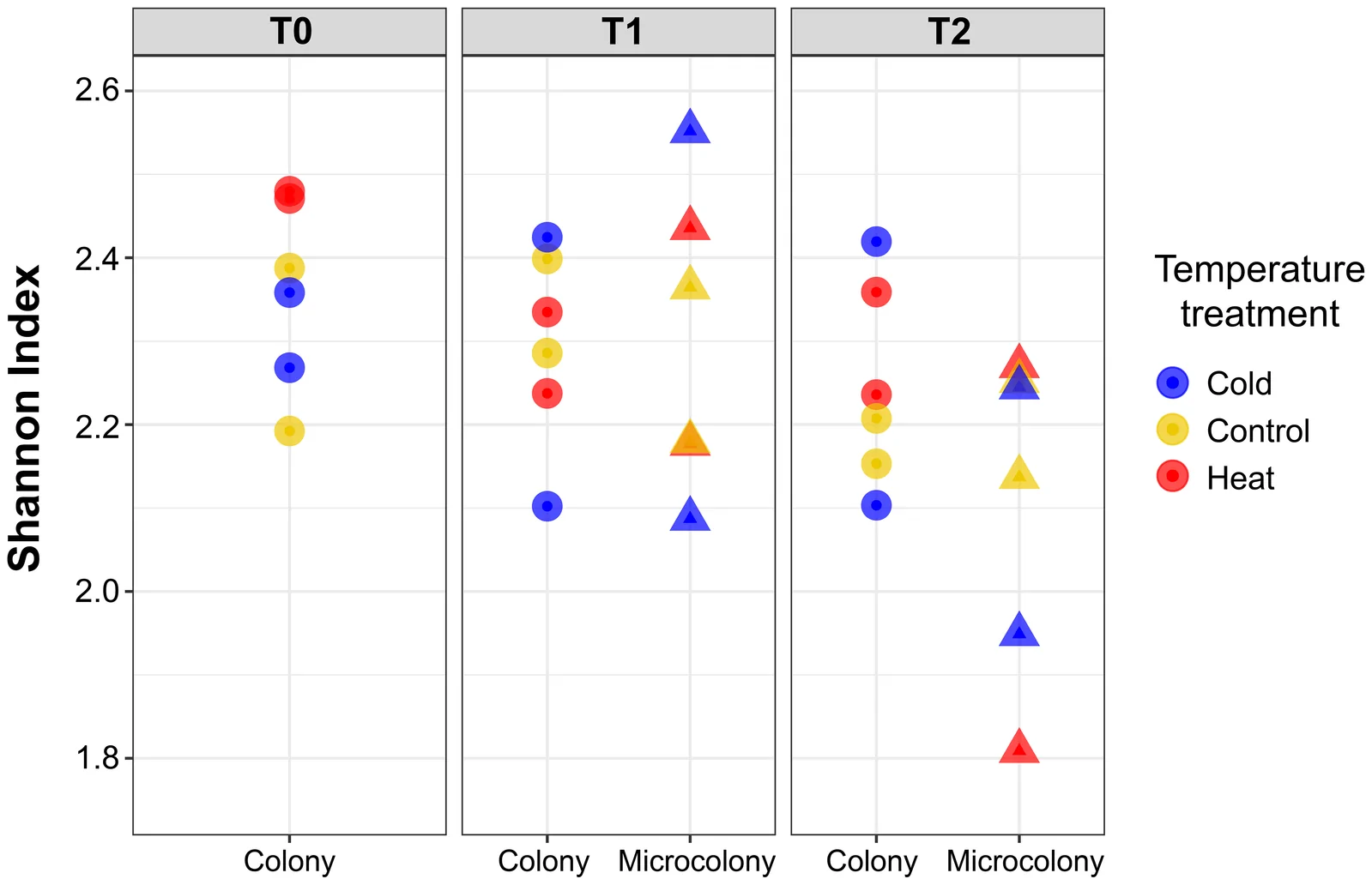
Microbiota alpha diversity measured with the Shannon index in workers from different temperature and colony settings. T0: time of collection before temperature stress treatments, T1: time of collection after five days of temperature stress treatments (9°C, 28˚C and 38°C), T2: time of collection after five days of recovery at 28°C.

Microbiota alpha diversity, measured with the Shannon index, was lowest in workers from microcolonies collected at the recovery phase from temperature stress treatments (T2, **Figure 2**). However, the difference in alpha diversity between workers from microcolonies and those from colonies in the recovery phase was not significant (Welch’s *t*-test, t = 1.47, df = 8.47, *p*-value = 0.175). The microbiota of workers from microcolonies collected during the stress treatments (T1) showed similar levels of alpha diversity to the microbiota of workers from colonies collected at all sampling time points (**Figure 2**).

**Figure 3:**
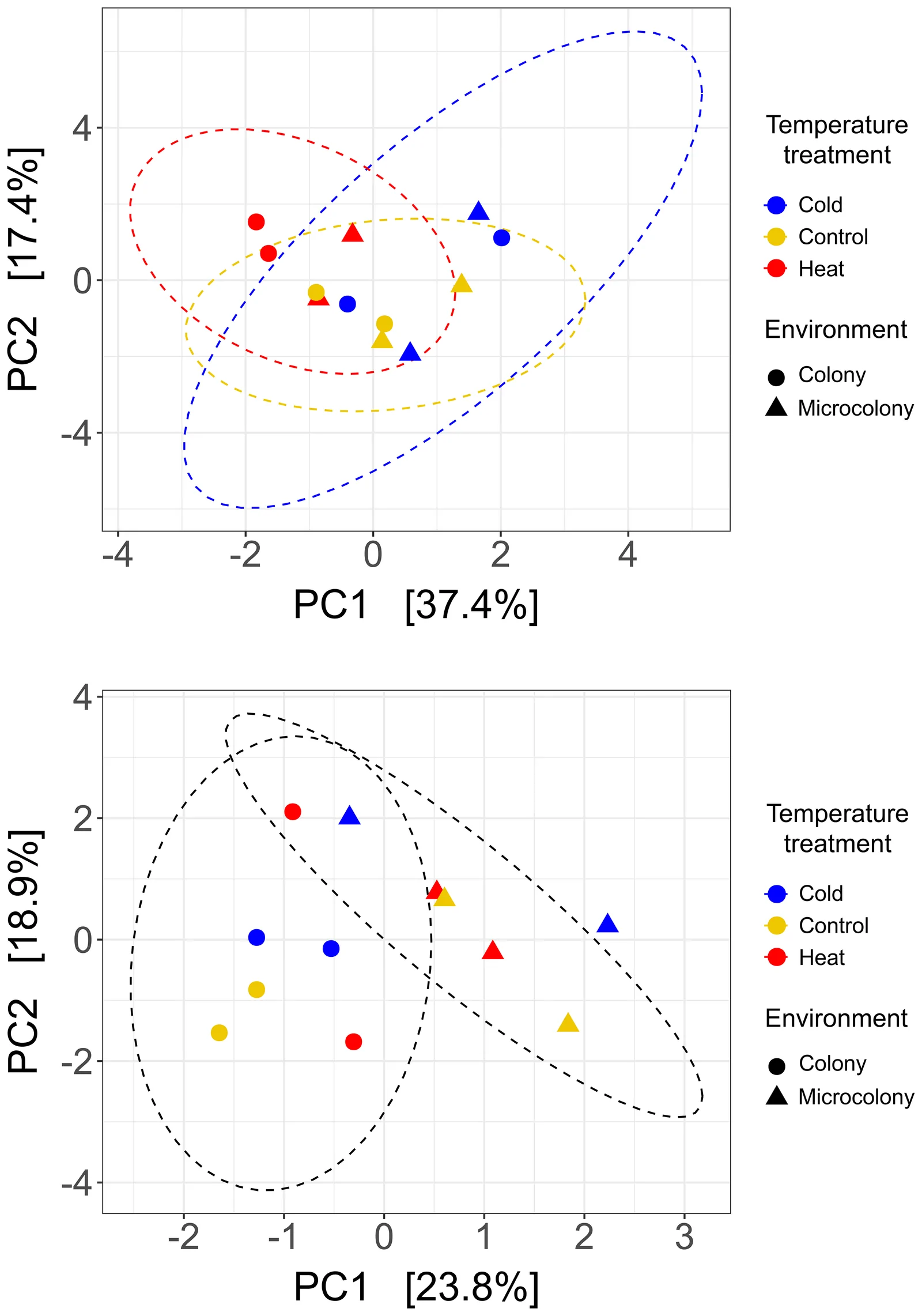
PCAs of microbial communities from workers in different temperature and colony settings. Read counts were transformed using the centered log-ratio (CLR) transformation. Upper figure shows the microbial communities from workers collected after five days of exposure to temperature stress treatments (T1). PERMANOVA results indicated a significant effect of the temperature treatment on gut microbiota composition (F = 2.16, R^2^ = 0.31, *p*-value = 0.026). Lower figure shows the microbial communities from workers collected after five days of recovery from temperature stress (T2). PERMANOVA results indicated a significant effect of the colony environment on gut microbiota composition (F = 2.37, R^2^ = 0.188, *p*-value = 0.007).

A two-way PERMANOVA carried out with workers in the control group indicated that the colony environment had a significant impact on microbiota composition (F = 3.72, R^2^ = 0.34, *p*-value = 0.003; **Supplementary Figure S2**) but the time of worker collection did not (F = 0.57, R^2^ = 0.10, *p*-value = 0.89). However, permutation tests indicated that the assumption of homogeneity of multivariate dispersion was not met for the colony environment variable. Two-way PERMANOVAs indicated that the temperature treatment had a significant effect on the microbiota composition when workers were under the temperature stress treatments (T1) but not when they were returned at 28°C (T2; **Table 1**; **Figure 3**). Pairwise adonis tests with workers from T1 indicated that the heat stress treatment (38°C) showed a higher effect than the cold stress treatment (9°C), although it was not significant (Heat vs Control: df = 1, F = 2.21, R^2^ = 0.269, adjusted *p*-value = 0.118; Cold vs Control: df = 1, F = 0.938, R^2^ = 0.135, adjusted *p-*value = 0.870*).* The colony environment significantly influenced the microbiota composition only in workers collected after the recovery phase (T2, **Table 1**; **Figure 3**).

**Table 1:** PERMANOVA analyses on microbiota composition under different temperature and colony settings.

Factor	df	F-value	R^2^	*p*-value
Two-way PERMANOVA with T1 workers				
**Temperature treatment**	**2**	**2.16**	**0.311**	**0.026**
Colony environment	1	1.55	0.112	0.155
Two-way PERMANOVA with T2 workers				
Temperature treatment	2	1.11	0.177	0.337
**Colony environment**	**1**	**2.37**	**0.188**	**0.007**

† Significant effects at the 95% level are highlighted in boldface. T1: time of collection after five days of temperature stress treatments (9°C, 28˚C and 38°C), T2: time of collection after five days of recovery at 28°C. The interaction term between the temperature treatment and the colony environment was not included in the two-way PERMANOVA as it was not significant in previous analyses (p-value > 0.05).

### 3.3 Differentially abundant taxa in workers exposed to heat and isolated from the colony

The ANCOM-BC identified a significant lower abundance of *Schmidhempelia* spp. (beta coefficient = -1.53, W = -0.92, *p*-value < 0.001), and a higher abundance of *Limosilactobacillus* spp. (beta coefficient = 3.21, W = 2.10, *p*-value < 0.001) in the gut of workers exposed to the heat treatments for five days (**Table 2**; **Figure 1**). These findings should be considered with caution, as comparisons were based on a limited sample size per group (n = 4).

**Table 2:** ANCOM-BC results of differentially abundant genera in the gut of workers exposed to heat compared to control workers, after five days of temperature treatments (T1).

Genus	Beta coefficient	W	*p*-value
*Ralstonia* spp.	-0.284	-0.741	0.890
***Limosilactobacillus* spp.**	**3.21**	**2.103**	**<0.001**
***Schmidhempelia* spp.**	**-1.528**	**-0.917**	**<0.001**
*Snodgrassella* spp.	0.106	0.301	0.890
unclassified Lactobacillaceae	-0.113	-0.240	0.890
*Bombilactobacillus* spp.	-0.025	-0.046	0.962
unclassified Orbaceae	-0.997	-0.968	0.890
*Gilliamella* spp.	0.225	0.451	0.890
*Apibacter* spp.	-0.977	-0.656	0.890
unclassified Bacteria	0.351	0.232	0.890
*Lactobacillus* spp.	0.208	0.479	0.890
unclassified Gammaproteobacteria	1.342	0.699	0.890

† Significant results at the 5% confidence level are highlighted in boldface. *P*-values were adjusted using the Benjamini-Hochberg correction.

In workers from microcolonies, the ANCOM-BC at the genus level identified a significantly higher abundance of *Schmidhempelia* spp. (beta coefficient = 1.91, *p*-value < 0.001) than in workers within the colony during the recovery phase from the temperature stress treatments (**Table 3**; **Figure 1**).

**Table 3:** ANCOM-BC results of differentially abundant genera in the gut of workers within microcolonies compared to workers within the colonies during the recovery phase from the temperature stress treatments.

Genus	Beta coefficient	W	*p*-value
*Ralstonia* spp.	0.464	0.950	0.512
*Limosilactobacillus* spp.	1.433	0.817	0.512
unclassified Bacteria	-0.634	-0.782	0.512
***Schmidhempelia* spp.**	**1.907**	**1.401**	**<0.001**
*Snodgrassella* spp.	-0.122	-0.297	0.830
*Bombilactobacillus* spp.	1.100	2.539	0.072
unclassified Orbaceae	0.960	1.220	0.481
*Gilliamella* spp.	0.045	0.119	0.905
*Lactobacillus* spp.	-0.923	-1.970	0.211
*Bacillus* spp.	-0.790	-0.795	0.512
*Apibacter* spp.	-1.333	-0.897	0.512
unclassified Lactobacillaceae	1.050	2.710	0.072
unclassified Gammaproteobacteria	-1.757	-1.549	0.393

† Significant results at the 5% confidence level are highlighted in boldface. *P*-values were adjusted using the Benjamini-Hochberg correction.

A further analysis carried out at the strain level indicated that five strains from the genus *Lactobacillus* and one *Gilliamella* strain were less abundant in the guts of workers from microcolonies, whereas one *Schmidhempelia* strain, one *Bombilactobacillus bombi* strain and three unclassified strains of the family Lactobacillaceae were more abundant in the guts of workers from microcolonies collected after the recovery phase (**TS1**).

## 4 Discussion

Workers from different colonies were exposed to different temperature treatments: cold (9°C), control (28°C) and heat (38°C), to study the effect of temperature stress on their gut microbiota composition. We placed some workers in microcolonies to analyze if the thermoregulation of the colony had a protective effect on the gut microbiota against environmental temperature changes. Samples were collected before the temperature stress treatments (T0), after five days of exposure to the different temperatures (T1), and after five days of recovery from the temperature stress at 28°C (T2).

### 4.1 Effect of heat and cold temperatures on worker gut microbiota composition

Temperature treatment significantly affected the microbiota composition of workers during exposure at different temperatures, although *post-hoc* comparisons did not reach statistical significance for either treatment. While the heat treatment showed a numerically higher effect than the cold treatment, this difference should be interpreted with caution given the lack of statistical significance. Based on previous studies, we expected a decrease in the relative abundance of core bacteria under cold stress. Bosmans et al. (33) studied the microbiota of queens before and after artificial hibernation and found that *Snodgrassella* and *Gilliamella* relative abundances drastically decreased, whereas non-core bacteria increased after hibernation and increased alpha diversity. *Snodgrassella* and *Gilliamella* seem more limited by cold than hot temperatures *in vitro* (23). Additionally, Hotchkiss et al. (54) ceased to detect *Snodgrassella* and *Schmidhempelia* in *B. impatiens* queens during late diapause. However, we did not find a decrease in core bacteria under cold temperatures nor a particular abundance of non-core bacteria. Our results on bumblebees agree with the findings by Ludvigsen et al. (29)Maes et al. (30) and Brar et al. (55) of stable gut microbiota compositions in honey bees throughout the winter. In fact, Ludvigsen et al. (29) observed that despite marked environmental temperature changes in winter, honey bees had similar midgut/pyloric microbiota when the diet was controlled. Additionally, other studies in honey bees have pointed to dietary changes, feces retention during winter, confinement inside the colony, and winter worker physiology as the factors responsible for the variation observed in microbiota throughout the seasons (25–28, 31, 32). Therefore, it is possible that the effect of the hibernation observed in Bosmans et al. (33) and Hotchkiss et al. (54) on the microbiota of bumblebee queens is explained by the metabolic changes associated with diapause rather than the environmental temperature *per se.* However, hibernation occurs for months and at lower temperatures than the one we used in our study, so we cannot discard an impact of cold temperature on the microbiota under those conditions.

Exposure to heat decreased the abundance of *Schmidhempelia* and increased the abundance of *Limosilactobacillus* in comparison with the control group. These ANCOM-BC results should be interpreted with caution given the small sample size per group (n = 4), which may limit the stability of differential abundance estimates. *Schmidhempelia* is a widespread genus strictly associated with bumblebees (56) and has been suggested to acidify the gut, potentially inhibiting parasites and facilitating colonisation by core symbionts (57). Its abundance was low compared with other core genera. *Limosilactobacillus* was represented in our study by only one strain identified as *L. reuteri*. Previously classified as *Lactobacillus reuteri*, this species is a non-core bacterium associated with vertebrate hosts including humans, but has also been isolated from the guts of *Apis* spp. (58). In *Apis cerana*, *L. reuteri* has been shown to improve immune function and worker survival and has been proposed as a probiotic (59). Both genera appeared at low relative abundances and without a clear pattern across samples. These observations may partly reflect the limited number of colonies included in our experiment, and further studies will be needed to confirm any temperature-related effects on these taxa.

Several studies have already pointed to the heat tolerance of bumblebee symbionts. *In vitro*, strains of *Snodgrassella*, *Gilliamella* and *Lactobacillus* can grow at temperatures of 40°C and show better growth rates at temperatures above the ones reached in bumblebee nests (23, 35, 36). In another study, Palmer-Young et al. (34) determined the effect of environmental temperature on the gut microbiota and the resistance to infection of *Bombus impatiens* at temperatures between 21°C and 37°C. Lactobacillaceae showed a twofold increase in total abundance across this range of temperature whereas the abundance of Neisseriaceae and Orbaceae bacteria slightly decreased but the Orbaceae change was not significant. However, we observed similar relative abundances for these families among the three temperature treatments. The different results might be explained by our measure of relative abundances instead of total abundances. Nonetheless, the stability in Lactobacillaceae relative abundance across a wider range of temperatures in our study seems better explained by a lower thermal response of the strains in comparison with those in the study of Palmer-Young et al. (2019).

The robustness of bumblebee gut microbiota composition under temperature stress can be explained by the need of the bacteria to adapt to a wide range of environmental temperatures. Within the bumblebee body they can be exposed to low temperatures when their host forage or the queens hibernate, and to hot temperatures when worker and queen bumblebees incubate larvae and pupae lying on top of the brooding structures and elevating their abdominal temperature. This has been pointed out as the most likely explanation for their heat tolerance and high optimal growth temperatures (23). Additionally, their horizontal transmission means both that they can be directly exposed to environmental temperatures during their *ex vivo* phases outside nests (60), and that they can undergo recombination and horizontal gene transfer (61), keeping their evolutionary potential to adapt to environmental conditions.

### 4.2 Effect of the colony environment on worker gut microbiota

Since worker gut microbiota composition remained stable through the different temperature treatments, we suggest that the colony thermoregulation does not protect the endosymbionts from the stress of environmental temperature changes as we expected. Nevertheless, we observed an effect of the colony environment on gut microbiota composition that was independent of the temperature treatments. We observed an overall decrease in alpha diversity in the microbiota of workers that had been placed in microcolonies for ten days. Specifically, five *Lactobacillus* strains and one *Gilliamella* strain decreased their abundance whereas one *Schmidhempelia* strain, one *Bombilactobacillus bombi* strain and three unclassified strains of Lactobacillaceae increased their abundance. However, microbiota composition was more variable among workers in microcolonies, and therefore differences between colony environments should be interpreted cautiously. Previous studies indicated that contact with the colony is required for bumblebees and honey bees to develop a complete gut microbiota (18, 19), and that workers with reduced contact with their colony exhibit lower relative abundances of *L. bombicola* and *B. bombi* (18). Our study suggests that contact with the colony also helps workers to maintain their gut microbiota composition. It is possible that a continuous ingestion of bumblebee endosymbionts within the colony is necessary to keep their microbiota diversity, and the limited contact with contaminated surfaces within the microcolonies would have caused the changes we observed.

In conclusion, our study suggests that worker gut microbiota composition is stable when workers are exposed to cold and hot temperatures for short periods of time. The microbiota of bees in microcolonies and intact colonies both showed little response to cold or heat. The robustness of the microbiota composition to environmental temperature changes might be explained by the adaptation of the endosymbionts to the wide temperature range they encounter both inside their host and during the *ex vivo* phases in which horizontal transmission occurs. This premise could be confirmed by studies involving more samples and longer periods of temperature stress. There is also a need to analyze how environmental temperature can affect the establishment of bumblebee microbiota, as colony thermoregulation might be necessary for the acquisition of complete microbiotas.

Finally, we observed that gut microbiota from workers within microcolonies was significantly different after ten days without contact with the origin colony showing a lower alpha diversity than the microbiota of workers within the colony. Therefore, although the colony environment did not play a role in protecting endosymbionts from environmental temperature changes, it may still provide bumblebees with continuous inocula that maintain the diversity of their microbiota.

This study has some limitations that should be considered when interpreting the results. First, the limited number of source colonies per treatment constrains the ability to capture the full extent of natural microbiome variability and reduces statistical power, a known challenge in microbiome research where small sample sizes can lead to reduced reproducibility and sensitivity to stochastic variation. Second, samples were analysed at the level of pooled worker guts, which precludes assessment of inter-individual variability. Third, the limited number of source colonies means that effects attributable to individual colony identity cannot be fully disentangled from treatment effects. Together, these factors suggest that the present results should be interpreted as reflecting patterns in the dominant taxa, with caution regarding their generalizability beyond the specific experimental conditions tested.

## Data Availability

The original contributions presented in the study are publicly available. This data can be found here: NCBI BioProject PRJNA1095982.
