# Supplementary material for: Short-term temperature fluctuations show minimal effects on worker gut microbiota in Bombus terrestris under controlled conditions
Source: Front Insect Sci. 2026 Aug 28;6:1867923. doi: 10.3389/finsc.2026.1867923 (PMC13562292; doi:10.3389/finsc.2026.1867923)
Supplement: Supplementary file 1 [file Supplementaryfile1.docx]

**Supplementary file**

**Figure S1**. Rarefaction curves from high-throughput sequencing. All samples reached saturation before 700 reads. Reads per sample ranged from 6,014 to 37,047.

**Figure S2.** PCA of microbial communities from workers in the control group. Read counts were transformed using the centered log-ratio (CLR) transformation. PERMANOVA results indicated a significant effect of colony environment on gut microbiota composition (F = 3.72, R2 = 0.34, *p*-value = 0.003). However, multivariate dispersion was also significantly different between workers within the colony and workers in microcolonies (*p*-value = 0.025).

**Table S1.** ANCOM-BC results of differentially abundant OTUs in the gut of workers within microcolonies compared to workers within the colonies during the recovery phase from the temperature stress treatments*. P*-values were adjusted using the Benjamini-Hochberg correction.

| **Genus** | **beta coefficient** | **W** | ***p*-value** |
| --- | --- | --- | --- |
| OTU1_*Ralstonia pickettii* | 0.434 | 1.116 | 0.480 |
| **OTU10_*Lactobacillus bombicola*** | **-1.888** | **-1.040** | <**0.001** |
| OTU101_Unclassified Snodgrassella | -1.639 | -1.609 | 0.355 |
| OTU106_Unclassified Lactobacillaceae | 1.707 | 1.475 | 0.390 |
| OTU11_Unclassified Lactobacillaceae | -2.018 | -1.333 | 0.447 |
| OTU114_Unclassified Lactobacillaceae | 0.980 | 1.027 | 0.480 |
| **OTU12_Unclassified Lactobacillus** | **-3.609** | **-3.157** | <**0.001** |
| OTU13_Unclassified Bombilactobacillus | -2.001 | -1.324 | 0.447 |
| OTU135_Unclassified Snodgrassella | 0.899 | 1.020 | 0.480 |
| OTU136_Unclassified Lactobacillus | -0.656 | -0.810 | 0.536 |
| OTU14_Unclassified Gilliamella | 0.289 | 0.230 | 0.867 |
| **OTU146_Unclassified Gilliamella** | **-2.361** | **-1.853** | <**0.001** |
| OTU15_*Limosilactobacillus reuteri* | 1.402 | 0.792 | 0.536 |
| OTU150_Unclassified Bacteria | -0.664 | -0.812 | 0.536 |
| **OTU16_Unclassified Schmidhempelia** | **1.876** | **1.277** | <**0.001** |
| OTU17_Unclassified Bombilactobacillus | -0.523 | -0.362 | 0.775 |
| **OTU18_Unclassified Lactobacillus** | **-3.831** | **-3.134** | <**0.001** |
| **OTU19_Unclassified Lactobacillaceae** | **4.105** | **3.221** | <**0.001** |
| OTU2_Unclassified Snodgrassella | -0.165 | -0.487 | 0.720 |
| **OTU20_Unclassified Lactobacillaceae** | **2.905** | **2.111** | <**0.001** |
| OTU21_Unclassified Lactobacillaceae | -0.534 | -0.369 | 0.775 |
| **OTU22_Unclassified Lactobacillus** | **-3.834** | **-3.123** | <**0.001** |
| OTU23_*Lactobacillus helsingborgensis* | -1.862 | -1.480 | 0.390 |
| **OTU24_Unclassified Lactobacillus** | **-3.638** | **-3.165** | <**0.001** |
| OTU25_Unclassified Lactobacillus | -0.803 | -0.469 | 0.720 |
| OTU26_Unclassified Lactobacillus | -0.801 | -0.590 | 0.653 |
| OTU27_Unclassified Lactobacillaceae | 1.081 | 1.115 | 0.480 |
| OTU28_*Bombilactobacillus bombi* | 1.764 | 1.626 | 0.355 |
| OTU29_Unclassified Lactobacillaceae | 1.081 | 1.115 | 0.480 |
| **OTU3_*Bombilactobacillus bombi*** | **1.181** | **3.085** | **0.017** |
| **OTU30_Unclassified Lactobacillaceae** | **2.574** | **2.249** | **0.000** |
| OTU31_Unclassified Lactobacillaceae | 1.828 | 1.631 | 0.355 |
| OTU32_Unclassified Lactobacillaceae | 1.015 | 0.758 | 0.540 |
| OTU34*_Lactobacillus melliventris* | -1.006 | -0.908 | 0.532 |
| OTU37_Unclassified Lactobacillus | 0.042 | 0.034 | 0.972 |
| OTU38_Unclassified Orbaceae | 0.929 | 1.144 | 0.480 |
| OTU4_Unclassified Gilliamella | 0.022 | 0.063 | 0.967 |
| OTU41_Unclassified Lactobacillaceae | 1.995 | 1.632 | 0.355 |
| OTU42_Unclassified Lactobacillaceae | 1.969 | 1.626 | 0.355 |
| OTU43_Unclassified Lactobacillus | 0.988 | 1.089 | 0.480 |
| OTU44_Unclassified Lactobacillus | 1.034 | 1.090 | 0.480 |
| OTU46_Unclassified Lactobacillus | -0.754 | -0.855 | 0.532 |
| OTU48_Unclassified Lactobacillaceae | 0.189 | 0.154 | 0.911 |
| OTU5*_Lactobacillus apis* | -2.445 | -2.201 | 0.183 |
| OTU51_Unclassified Bacillus | -0.820 | -0.872 | 0.532 |
| OTU6*_Apibacter mensalis* | -1.363 | -0.888 | 0.532 |
| OTU65_*Bombilactobacillus mellis* | -0.765 | -0.858 | 0.532 |
| OTU7_Unclassified Lactobacillus | 0.960 | 0.780 | 0.536 |
| OTU75_Unclassified Lactobacillus | 0.885 | 1.018 | 0.480 |
| OTU8_*Lactobacillus panisapium* | -2.342 | -1.323 | 0.447 |
| OTU86_Unclassified Snodgrassella | 1.717 | 1.516 | 0.390 |
| OTU9_Unclassified Lactobacillaceae | 1.291 | 1.186 | 0.480 |
| OTU95_Unclassified Gammaproteobacteria | -1.788 | -1.617 | 0.355 |
